# Potent Intestinal Mucosal Barrier Enhancement of *Nostoc commune* Vaucher Polysaccharide Supplementation Ameliorates Acute Ulcerative Colitis in Mice Mediated by Gut Microbiota

**DOI:** 10.3390/nu15133054

**Published:** 2023-07-06

**Authors:** Min Guo, Dehai Xing, Jin Wang, Ying Zhang, Zhuoyu Li, Xiangying Jiao

**Affiliations:** 1 Department of Physiology, Key Laboratory of Physiology of Shanxi Province, Key Laboratory of Cellular Physiology of Ministry of Education, Shanxi Medical University, Taiyuan 030001, China; guom2020@sxmu.edu.cn (M.G.); xingdeh@163.com (D.X.); wangjin5680816@126.com (J.W.); 2 Department of Nutrition and Food Hygiene, School of Public Health, Peking University, Beijing 100191, China; 2011110189@bjmu.edu.cn; 3 Institute of Biotechnology, Key Laboratory of Chemical Biology and Molecular Engineering of Ministry of Education, Shanxi University, Taiyuan 030006, China

**Keywords:** mucosal barrier, gut microbiota, ulcerative colitis, polysaccharides

## Abstract

Ulcerative colitis (UC) is evolving into a global burden with a substantially increasing incidence in developing countries. It is characterized by inflammation confined to mucosa and is recognized as an intestinal barrier disease. The intestinal microbiota plays a crucial role in UC pathogenesis. *N. commune* has long been appreciated as a healthy food and supplement worldwide and polysaccharides account for 60%. Here, we examined the amelioration of *N. commune* polysaccharides against acute colitis in mice induced by DSS and assessed the mediating role of gut microbiota. An integrated analysis of microbiome, metabolomics, and transcriptomics fully elaborated it markedly enhanced intestinal mucosal barrier function, including: increasing the relative abundance of *Akkermansia muciniphila, uncultured_bacterium_g__norank_f__Muribaculaceae*, and *unclassified_g__norank_f__norank_o__Clostridia_UCG-014*; decreasing microbiota-derived phosphatidylcholines and thromboxane 2 levels mapped to arachidonic acid metabolism; improving mucin2 biosynthesis and secretion; enhancing ZO-1 and occludin expression; reducing neutrophil infiltration; regulating the level of colitis-related inflammatory cytokines; involving inflammation and immune function-associated signaling pathways. Further, the mediation effect of gut microbiota was evaluated by administering a cocktail of antibiotics. In conclusion, our results demonstrated that *N. commune* polysaccharides predominantly reinforced the gut microbiota-mediated intestinal mucosal barrier to confer protection against UC and exhibited dramatic prebiotic-like functions, providing an alternative or complementary treatment for UC.

## 1. Introduction

Ulcerative colitis (UC) is one subtype of inflammatory bowel disease (IBD) [1,2]; it is a growing concern worldwide due to its high prevalence in developed nations and the significant rise in occurrences in developing countries [1,3]. It is characterized by inflammation confined to the mucosa, beginning in the rectum and spreading proximally in the colon [1,2,4]. Although the precise cause of UC remains unclear, various factors, such as an imbalanced immune response, modified gut microbiota, hereditary predisposition, and external factors, have been suggested as possible contributors [1,5].

UC is an intestinal barrier disease caused initially by intestinal epithelial dysfunction [1,6]. Many structural components maintain a fragile balance to sustain the homeostasis of the intestinal barrier [1]. The intestinal microbiota plays a significant role in improving barrier function by providing nutritional and dietary factors on the luminal surface [1,7]. The first layer of protection is formed by the underlying epithelium and the mucus layer that follows the intestinal lumen [8,9,10]. Lamina propria is the innermost layer, which maintains the non-inflamed condition of the tissue [1,11]. Intestinal barrier deficiencies have been associated with a diverse range of diseases, thus indicating a novel therapeutic objective [7,12].

The intricate nature of the pathogenesis of UC suggests that the interaction between the host and intestinal microbiota is widely acknowledged as a pivotal factor [5]. The disturbance of the microbiota leads to a rapid proliferation of pathogenic bacteria within the intestinal tract directly invading and damaging the epithelial cells and intestinal mucosal barrier [5,13]; intestinal mucosal barrier function declines, the shield function of the intestinal wall abates, and the translocation of the intestinal microbiota exacerbates the damage to the intestinal mucosal barrier, leading to a detrimental cycle and intensifying the inflammatory response within the intestines [5,14]. In addition, gut microbiota-derived metabolites secreted into the lumen directly interact with epithelium and immune cells and significantly influence the preservation of gut barrier integrity and the maintenance of intestinal homeostasis [15,16,17].

Currently available treatments for UC, including aminosalicylic acid, corticosteroids, immunomodulators, monoclonal antibodies, and even surgeries, often have side effects, drug resistance, and poor clinical efficacy [2,18]. Hence, it is imperative to undertake an immediate investigation into a novel, secure, and efficacious strategy for patients with UC. Natural bioactive polysaccharides have attracted increasing attention for their exceptional advantages of safety, accessibility, and good biocompatibility [19,20,21]. It is well established that most of them can be metabolized by the gut microbiota and can exert profound protection against oxidative stress, toxins, inflammatory responses, and tumors [22]. Although the protection of natural polysaccharides against colitis has been recently extensively studied, the role of gut microbiota and gut microbiota-derived metabolite remains incompletely understood and confirmed.

*Nostoc commune* Vaucher. (*N. commune*) is classified as a scarce macroscopic cyanobacterium and has long been appreciated worldwide as a healthy food and medicine [23]. Owing to the noticeable phytophysiological stress property, the protective physiological and pharmacological activity of it and its extracts are of particular interest and have promising characteristics. The polysaccharides from it have been shown to have some favorable characteristics, including being identified as heteropolysaccharides, with an ultra-high molecular weight of 210 kDa, as well as accounting for up to 60% of the dry weight of *N. commune* [24], suggesting fairly compelling bioactivity. We, therefore, aimed to elucidate whether the polysaccharides were protective against the occurrence of UC and to understand and explain the role of gut microbiota. An integrated analysis of multi-omics data, including the microbiome, non-targeted metabolomics, and transcriptomics, with the macroscopic and histopathological examination, fully elaborated that the polysaccharides markedly attenuated intestinal mucosal barrier damage, which mainly compromises UC. Together, our results revealed that the polysaccharides from *N. commune* predominantly strengthened the gut microbiota-mediated intestinal mucosal barrier to confer protection against UC in mice and exhibited dramatical prebiotic-like functions, offering an alternative or supplementary therapeutic approach for individuals diagnosed with IBD, especially UC.

## 2. Materials and Methods

### 2.1. Mice

All research conducted on mice was in accordance with and approved by the institutional Animal Ethics Committee at Shanxi Medical University. Male C57BL/6J mice were obtained from Beijing Vital River Laboratory Animal Technology Co., Ltd. (Beijing, China) as mixed littermates and housed under SPF and pathogen-free conditions in the animal facility at the Yingze Campus Laboratory Animal Research Center of Shanxi Medical University. The mice were co-housed for a duration of one week prior to being randomly assigned to experimental groups in order to synchronize the gut microbiome across the individual mice and to minimize heterogeneity. The investigators did not employ blinding techniques for allocation during experiments and outcome assessment, unless specified sections explicitly incorporated blind assessment.

### 2.2. Induction of Acute DSS Colitis and Intervention

**Figure 1:**
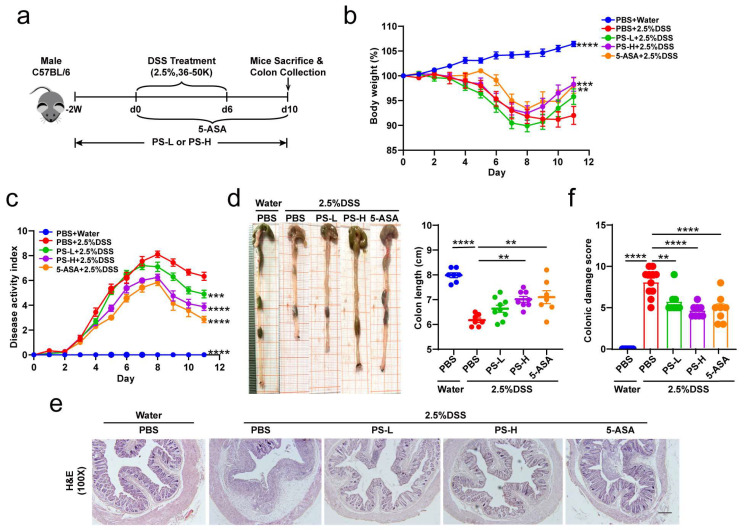
Oral PS attenuates DSS-induced colitis. (**a**) Schematic diagram illustrating the experimental system: C57BL/6 mice received a daily oral gavage of normal saline or low dose of PS (PS-L) or high dose of PS (PS-H) starting 2 weeks before being subjected to dextran sodium sulfate (DSS)-induced colitis and sustained until the end of the experiment. Mice were treated with 2.5% DSS in drinking water ad libitum for 6 days; 5-aminosalicylic acid (5-ASA) was served as a positive control and was administered on the day that DSS treatment started. n = 16 mice per group. (**b**) Daily mean body weight changes in each group described in (**a**). n = 7–10 mice per group. (**c**) Changes in DAI score (composited score of body weight, bleeding, and stool consistency) after water or DSS oral treatment. n = 7–10 mice per group. (**d**) Representative colons from each group described in (**a**) and statistical analyses for colon length. n = 7–9 mice per group. Representative photomicrographs (100×) of colon tissue staining by H&E ((**e**), scale bar = 200 μm) and quantitative analyses for colonic damage scores ((**f**), n = 7–9 mice per group). ** *p* < 0.01, *** *p* < 0.001, **** *p* < 0.0001 as determined by one-way ANOVA (**b**–**e**). Data represent mean ± SEM.

The model was conducted following a reported protocol with some minor modifications [25,26] (Figure 1a). Briefly, mice received 2.5% dextran sodium sulfate (DSS, 36–50 kDa; MP Bio, Santa Ana, CA, USA) supplemented in the drinking water for 6 days, followed by normal water. PS was pre-administered at 200 mg/kg (PS-L) or 400 mg/kg (PS-H) by daily intragastric gavage that was started 2 weeks before being subjected to DSS-induced colitis and was sustained until the end of the experiment. The preparation and characteristics of polysaccharides from *N. commune* (PS) were analyzed and described in our previous article [24]. 5-aminosalicylic acid (5-ASA) was used as a positive control. Control healthy mice were exclusively given regular water. In some experiments, we pretreated mice by administering a cocktail of antibiotics (vancomycin 0.5 g/L, ampicillin 1.0 g/L, metronidazole 1.0 g/L, and neomycin 1.0 g/L) in drinking water [27] for 4 weeks and replaced with a freshly prepared cocktail once every second day before DSS induction. Changes in body weight were determined daily over the 10 ds experimental period. Evaluation of disease activity index (DAI) was conducted by combining the parameters of weight loss, stool consistency, and rectal bleeding, as described previously [28,29]. The mean of the total score of the three parameters was calculated as the DAI. The sample of colonic content was collected for gut microbiome and metabolomics analysis. The colon was carefully isolated and the length was measured. Then, the proximal colon was kept for MPO (frozen immediately), the middle portion for RNA and protein isolation, and the rectal region for histological assessment and immunofluorescence staining [26].

### 2.3. Colonic Histopathology

Colon segments were fixed in 4% paraformaldehyde for 24 h and embedded in paraffin. H&E staining was performed to evaluate colon pathological injury. The same rectal region of the colon from different groups was compared to analyze colitis severity. Histological damage was scored for epithelial damage and inflammatory cell infiltration in a blinded fashion. AB staining was performed to visualize the mucins. Data were analyzed using ImagePro Plus software (Version 6.0, Media Cybernetics, Rockville, MD, USA).

### 2.4. MPO Activity Measurement

Colonic MPO activity was measured by Myeloperoxidase Assay Kit (Jiancheng Bioengineering Institute, Nanjing, China) according to the manufacturer’s instructions.

### 2.5. Immunohistochemistry

Antigens were recovered by treatment of samples at 98 °C for 10 min in 10 mM citrate buffer (pH 6.0). For immunohistochemical analysis, endogenous peroxidase was blocked with 10% H_2_O_2_ for 20 min and nonspecific antigens were blocked with serum for 30 min at room temperature. The slides were then incubated with specific primary antibodies at 4 °C for 12 h. Antibodies used were: MUC2 (1:2000, Proteintech, Wuhan, China), Ly-6 g (1:80, Proteintech), and F4/80 (1:2000, Proteintech). Slides were then treated with HRP-conjugated secondary antibodies. Expression of the stained markers was calculated using a histochemistry score (H-SCORE = 3x% of strongly staining cells + 2x% of moderately staining cells + 1x% of weakly staining cells).

### 2.6. Full-Length 16S rRNA Gene Sequencing

The DNA was extracted from the colonic content samples using a QIAamp DNA Isolation Kit (Qiagen, Hilden, Germany). Nanodrop 2000 was used to check the quantity and quality of the extracted DNA. The full-length bacterial 16S rRNA gene was amplified with primer pairs: 27F (5′-AGAGTTTGATCMTGGCTCAG-3′) and 1492R (5′-GGTTACCTTGTTACGACTT-3′) by an ABI GeneAmp 9700 PCR thermocycler (ABI, Los Angeles, CA, USA). Purified amplicons were pooled in an equimolar and a DNA library was constructed using the SMRTbell Express Template Prep Kit 2.0 (PacBio, Menlo Park, CA, USA). Purified SMRTbell libraries were sequenced on a Pacbio Sequel II System (PacBio, Menlo Park, CA, USA) according to the standard protocols by Majorbio Bio-Pharm Technology (Shanghai, China). The raw 16S rRNA gene sequencing reads were demultiplexed to circular consensus sequence (CCS) reads by SMRTLink version 8.0 and length-filtered (<1000 or >1800 bp). Operational taxonomic units (OTUs) with 97% similarity cutoff were clustered using UPARSE version 7.1; chimeric sequences were identified and removed. The taxonomy of each OTU representative sequence was analyzed by RDP Classifier version 2.11 against the 16S rRNA database (Silva v138) with a confidence threshold of 0.7. All obtained raw sequence datasets have been uploaded to the NCBI Sequence Read Archive (SRA) with the accession number PRJNA988420.

### 2.7. LC-MS Metabolomic Analysis Technology

Each 50 mg colon tissue sample was added to 400 μL of pre-cooled methanol–water solution (1:1, v/v), precipitated, disrupted, and centrifuged. The supernatant was collected for mass spectrometry analysis. Samples were separated by a Thermo Fisher Scientific Vanquish Horizon system. To mitigate the influence of fluctuations in instrument detection signals, a sequential analysis of samples was conducted. Within the sample queue, a quality control (QC) sample was interspersed at regular intervals among every 10 experimental samples; it was applied to assess and appraise the system’s stability and the dependability of the experimental data. Electrospray ionization (ESI) was employed for the identification and quantification of both positive and negative ions. Samples were separated by UHPLC and analyzed by Q-Exactive HF-X mass spectrometer (Thermo Fisher Scientific, Waltham, MA, USA). The original data were processed by Progenesis QI software (Version 2.3, Waters Corporation, Milford, CT, USA) and exported as a three-dimensional data matrix in CSV format. Internal standard peaks, as well as any known false positive peaks and the redundancy, were removed. Peaks were merged and HMDB (http://www.hmdb.ca/ (accessed on 1 January 2019)), Metlin (https://metlin.scripps.edu/ (accessed on 1 January 2017)), and Majorbio Database were retrieved. The extracted data from databases were uploaded to the Majorbio cloud platform (https://cloud.majorbio.com (accessed on 1 September 2016)) and preprocessed for missing value deletion and recoding, normalization, QC samples RSD assessment, and log10 logarithmization. The data matrix was by performed variance analysis; then, R package ropls (Version 1.6.2) was used to carry out multidimensional statistical analysis, including unsupervised principal component analysis (PCA) and orthogonal least partial squares discriminant analysis (OPLS-DA). Metabolites based on variable importance in the projection (VIP) and the p-value of the Student’s *t*-test were identified as differential metabolites (VIP > 1, *p* < 0.05). KEGG (http://www.genome.jp/kegg/ (accessed on 18 September 2021)) database was retrieved for metabolic pathway enrichment of differential metabolites between two groups. Scipy (Python packages, Version1.0.0) was employed to detect statistically significantly enriched pathways utilizing Fisher’s exact test.

### 2.8. Transcriptome Data Processing and Analysis

Total RNA underwent processing using Agilent 2100 Bioanalyzer in accordance with the manufacturer’s instructions (Agilent, Santa Clara, CA, USA). cDNA quantity and labeling efficiency were assessed using Agilent 2100 Bioanalyzer and ABI StepOne Plus Real-Time PCR and sequenced using the Illumina NovaSeq 6000 platform. The raw reads were filtered using the filter software fastq (Version 0.19.5); the comparison of gene expression in the RNA seq reads was performed using the HiSat2 software (Version 2.1.0). The reconstruction of transcripts for each sample was accomplished using StringTie (Version 2.1.2). Transcripts per million reads (TPM) were used to calculate gene expression levels. RSEM (Version 1.3.3) was used to quantify gene abundances. The DESeq2 software (Version 1.24.0) was utilized for differential expression analysis.

### 2.9. RNA and Protein Preparation

Colonic RNA and protein were extracted by E.Z.N.A. DNA/RNA/Protein Kit (Omega Bio-Tek, Guangzhou, China), providing a rapid and easy method for the isolation of total RNA, genomic DNA, and protein from animal tissues and allowing single processing of multiple samples in less than 40 min.

### 2.10. Western Blotting

Proteins were extracted from colon tissues and then quantitated using a Super-Rapid Protein Quantification Kit (Abbkine Scientific, Wuhan, China). Equal amounts of protein (25 μg) from different samples were separated by 10% SDS-PAGE and transferred to PVDF membranes. The membranes were blocked for 1 h at room temperature in blocking buffer (5% skimmed milk or 5% BSA in TBST), then probed with ZO-1 rabbit monoclonal Ab (1:2000, Affinity Biosciences, Changzhou, China), occludin rabbit monoclonal Ab (1:2000, Proteintech). Mouse monoclonal anti-β-actin Ab (1:10,000, Bio World, Nanjing, China) was used as the loading control. Images were captured using the ChemiDoc MP imaging system (Bio-Rad, Hercules, CA, USA). Blotted bands were quantified with Image J software (Version, 1.8.0.112, NIH).

### 2.11. ELISA

Serum pro- and anti-inflammatory cytokines (TNF-α/IL-1β/IL-6/TGF-1β/IL-10) were measured by a Mouse High Sensitivity ELISA Kit (MULTISCIENCES, Hangzhou, China) according to the manufacturer’s instructions.

### 2.12. Quantification and Statistical Analysis

Mice were assigned at random to each group for all mouse studies. Immunostaining and histological scoring were performed in a blinded fashion. Group sizes of 3 mice or above were sufficient to reach a statistical power of at least 80%. The data were first analyzed for normal distribution. Statistical significance was determined by using unpaired Student’s *t*-tests (comparison of two groups), Mann–Whitney tests (comparison of two groups, non-parametric data), or one-way ANOVA (comparison of three or more groups), followed by Bonferroni’s post hoc test. * *p* < 0.05, ** *p* < 0.01, *** *p* < 0.001, **** *p* < 0.0001. Data conforming to normal distribution were presented as mean ± SEM and statistical tests were carried out using GraphPad Prism software version 8.0.

## 3. Results

### 3.1. Oral PS Attenuates DSS-Induced Colitis

Excluding production batch errors by preliminary experiment, 2.5% DSS was administered to induce experimental acute colitis in the mice model. To explore the efficacy of PS against DSS-colitis, we orally gavaged male C57BL/6 mice with or without either low or high dose of PS (PS-L or -H) for 2 weeks before being subjected to DSS-associated injury (Figure 1a). During the induction of colitis, the addition of PS demonstrated a significant protective effect on mice, as shown by the prevention of DSS-induced body weight loss (Figure 1b), reduction in disease activity index (DAI) scores (Figure 1c), and mitigation of colon length shortening (Figure 1d). A readily observed pathological histological damage in the distal colon was seen, such as the malformation of crypt structure, disruption to shedding of the epithelial cell, and pronounced inflammatory cell infiltration in lamina and submucosa. However, the degree of injury was notably reduced in the groups that received PS pretreatment in comparison with the untreated group, with PS-H pretreatment demonstrating greater efficacy than PS-L (Figure 1e,f). These results suggested that PS administration led to less ulcerative colitis syndrome and showed superior efficacy in protecting against DSS-associated colitis.

### 3.2. Pretreatment with PS Mitigates the Dysbiosis of Gut Microbiota Induced by DSS-Colitis

**Figure 2:**
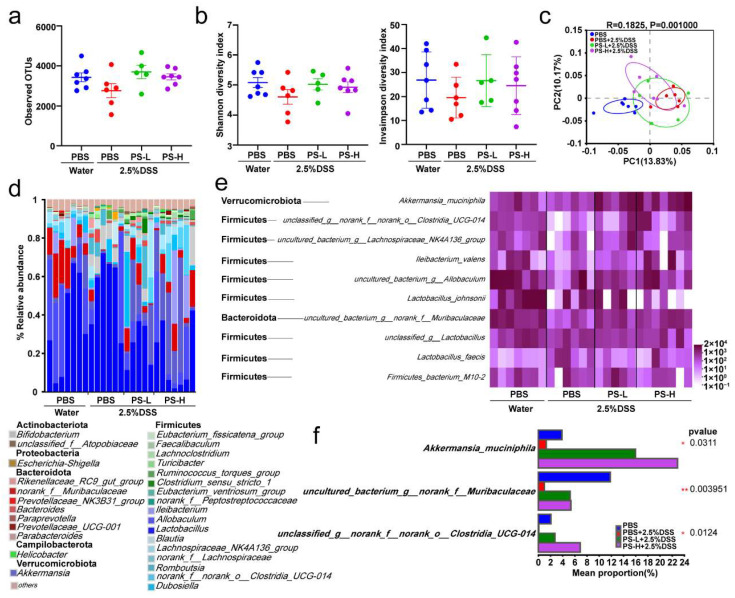
Pretreatment with PS mitigates gut microbial dysbiosis induced by DSS. (**a**) 16S rRNA sequencing analysis of colonic content samples in each group of mice described in Figure 1a. The graph depicts estimation of microbial community observed OTU (operational taxonomic unit) richness. (**b**) Shannon and inverse Simpson α-diversity index of grouped data. (**c**) PCoA of the gut microbiome composition on the genus level based on the unweighted_UniFrac_full_tree matrix for each group as described in Figure 1a (ANOSIM, *R* = 0.1825, *p* = 0.001). The shapes and colors of the spots serve as indicators for the samples obtained from each individual. Colored ellipses are utilized to represent the 0.95 confidence interval (CI) ranges within each tested group. (**d**) Relative abundance of gut bacterial phylum and genus in each group is depicted in Figure 1a. (**e**) Heatmap of the relative abundance of representative OTUs at the species level (top 10 of the total abundance) in mice. (**f**) Multiple comparisons of select species among each group (Kruskal–Wallis rank sum test followed by fdr test). *X*-axis indicates the mean proportion of relative abundance. The differences in the α-diversity index were determined by Student’s *t*-test (**a**,**b**). n = 5–7 mice per group (**a**–**f**).

It is widely believed that microbial dysbiosis is a critical pathogenic cause of UC. There is much consensus on the utilization of natural polysaccharides by gut microbiota to perform effective protection against oxidative stress, toxins, inflammatory responses, and tumors [22]. At the outset, the gut microbial community was first detected by full-length 16S rRNA gene sequencing analysis. We found that either the uninduced group or the pretreatment with PS group showed no difference with the DSS-induced colitis group in bacterial richness (observed OTU richness, Figure 2a) or alpha diversity (Shannon and inverse Simpson index, Figure 2b). PCoA based on unweighted UniFrac distance was used to further analyze the beta diversity of microbial composition. Significant variations in the beta diversity of gut microbiome signatures were observed between the group with DSS-induced colitis and the uninduced group. However, the beta diversity of DSS-induced colitis mice pretreated with PS exhibited a closer resemblance to that of the uninduced group, as depicted in Figure 2c. Classified by phylum levels, Figure 2d visualized the microbiome community composition in mice of each group based on genus level. *Bacteroidetes* and *Firmicutes* were the most dominant phylum in each group of mice, whereas, in the PS-pretreated groups, there was a higher proportion of the *Verrucomicrobiota* population (Figure 2d and Figure S1). Further analysis of the top 10 taxa of the total relative abundance at the species level found that, compared with the DSS group, PS pretreatment dramatically increased the relative abundance of *A. muciniphila* (known to regulate gut barrier functions), *uncultured_bacterium_g__norank_f__Muribaculaceae*, and *unclassified_g__norank_f__norank_o__Clostridia_UCG-014* (Figure 2e,f), which are all important microbial factors that are distinctively decreased in IBD patients [5]. These data presented suggest that the disruption of gut microbial dysbiosis caused by DSS can be restored through pretreatment with PS.

### 3.3. PS Pretreatment Altered Gut Microbiota-Related Metabolites in Colonic Contents

**Figure 3:**
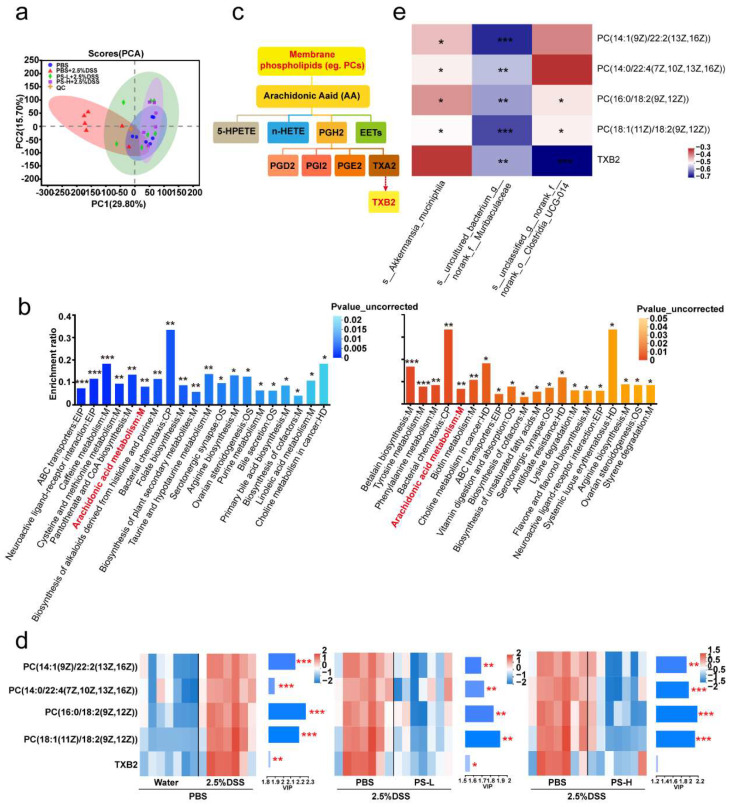
PS pretreatment altered gut microbiota-related metabolites in colonic contents. (**a**) Score scatter plot from PCA model of intestinal contents samples in each group of mice described in Figure 1a. n = 6–7 mice per group. (**b**) Histogram plot of KEGG metabolic pathway enrichment analysis for group 2.5%DSS + PBS versus water + PBS or 2.5%DSS + PS-L/H versus 2.5%DSS + PBS intestinal contents from (**a**). The color depth and column height indicate uncorrected *p*-value and the impact of the pathway. (**c**) Overview of arachidonic acid metabolism pathway. (**d**) VIP-score analysis of differential metabolites enriched in the arachidonic acid metabolism pathway for group 2.5%DSS + PBS versus water + PBS, 2.5%DSS + PS-L or 2.5%DSS + PS-H versus 2.5%DSS + PBS. (**Left**) Metabolite-clustering dendrogram: the color depth indicates the relative expression. (**Right**) Metabolite VIP bars: the bar length signifies the extent to which the metabolite contributes to the disparity between the two groups; the bar color depth calculated from -log10 (*p*-value) indicates the significance of difference (* *p* < 0.05, ** *p* < 0.01, *** *p* < 0.001). (**e**) Correlation analysis for selected differential metabolites showed in (**d**) with differential species obtained in Figure 2f.

Recent studies have shown that the microbiota could impact the pathogenesis of inflammatory bowel disease (IBD) through the production of metabolites, which are recognized as significant mediators in the interaction between the host and microbes [30,31]. Subsequently, the UHPLC-MRM-MS/MS analytical method was employed to ascertain alterations in fecal metabolites within the colonic contents of mice, resulting in the identification of a total of 2908 metabolites. Based on the OPLS-DA score chart, a notable distinction was observed between the metabolites of the DSS-induced colitis group and the remaining three groups (Figure 3a). Further, we analyzed the significantly differential metabolites between the selected two groups (*p* < 0.05, VIP value from OPLS-DA >1, and fold change >1): DSS-induced group versus uninduced group, 1217 significant differential metabolites, termed set 1; PS-L pretreatment group versus untreated DSS-colitis group, 1115, termed set 2; PS-H pretreatment group versus untreated DSS-colitis group, 1237, termed set 3. Due to a large number of significantly differential metabolites between each PS pretreatment group and untreated DSS-colitis group, the intersection of them was further analyzed and termed set 4 (Figure S2). Subsequently, these significantly differential metabolites in set 1 and 4 were, respectively, subjected to KEGG pathway enrichment analysis. We prioritized statistically significant differences over enrichment degree and found these metabolites converged on a shared arachidonic acid (AA) metabolism pathway (Figure 3b,c). Next, we focused on the metabolites mapped to AA metabolism (Figure 3d). A heatmap of hierarchical clustering analysis showed that the expression of phosphatidylcholines (PCs), including PC(16:0/18:2(9Z,12 Z)), PC(18:1(11Z)/18:2(9Z,12Z)), PC(14:0/22:4(7Z,10Z,13Z,16Z)), PC(14:1(9Z)/22:2(13Z,16Z)), and thromboxane (TXB) 2, were significantly lower in the colonic contents of mice that received PS pretreatment compared with the DSS-colitis group without PS pretreatment; the VIP value indicated the importance of significantly differential metabolites (Figure 3d). To determine the potential association of intestinal microbiota with metabolites, we further performed correlation analysis between differentially enriched microbes and metabolites by Spearman correlation. We observed that *unclassified_g__norank_f__norank_o__Clostridia_UCG-014* showed a profound negative correlation with the TXB2 level (*R* < −0.68, *p* < 0.001). *Uncultured_bacterium_g__norank_f__Muribaculaceae* exhibited a significantly negative correlation with the level of all differential metabolites (*R* < −0.54, *p* < 0.01). *A. muciniphila* had a strong negative correlation with the PC level (*R* < −0.40, *p* < 0.05) (Figure 3e). Taken together, we concluded that PS administration conduced to the reduction of colonic PCs and TXB 2 biosynthesis, which could potentially provide a protective mechanism against DSS-induced colon damage.

### 3.4. Pretreatment with PS Alleviated Intestinal Mucosal and Epithelium Barrier Damage and Reduced Inflammation Aroused by DSS-Colitis

**Figure 4:**
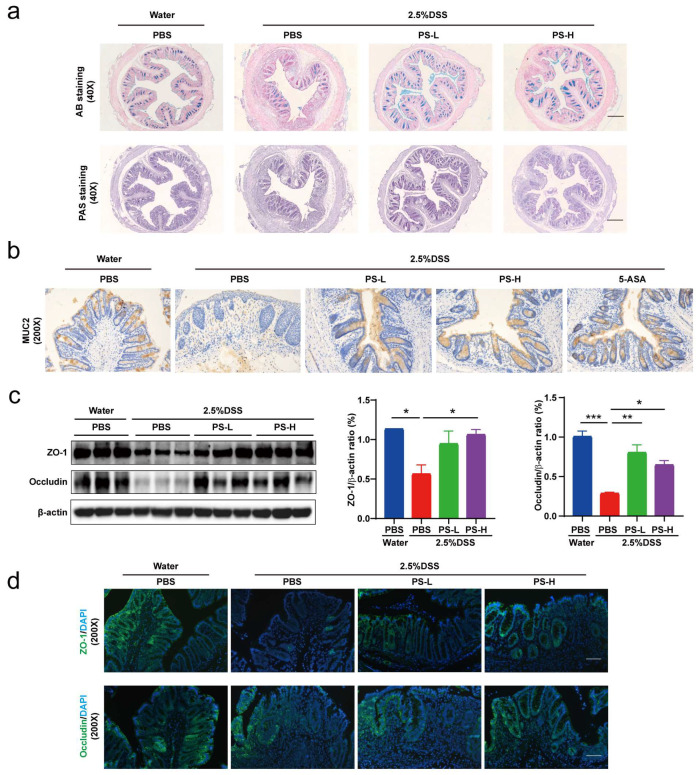

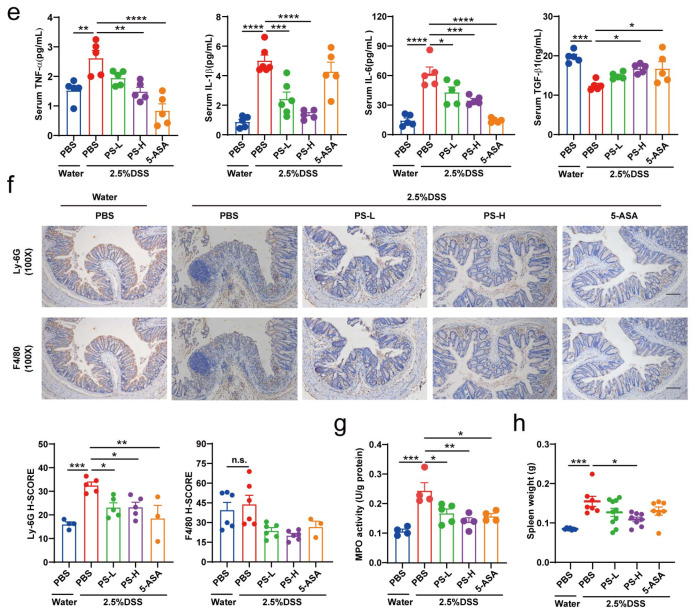
Pretreatment with PS alleviated intestinal mucosal and epithelium barrier damage and reduced inflammation aroused by DSS-colitis. (**a**) Representative photomicrographs (40×) of colon tissue staining by Alcian blue (AB) and periodic acid-Schiff (PAS). Scale bar = 500 μm. (**b**) Representative images for MUC2 immunohistochemistry staining (200×) in colon. Scale bar = 100 μm. (**c**) Western blot bands and quantification of ZO-1 and occludin-1 in representative samples. n = 3 mice per group. (**d**) Representative images (200×) for immunofluorescence labeled ZO-1 and occludin-1 in colon. Scale bar = 100 μm. (**e**) Concentrations of serum pro- and anti-inflammatory cytokines of each group. n = 5–6 mice per group. (**f**) Representative images for Ly-6 g and F4/80 immunohistochemistry staining (100×) in colon. n = 3–6 mice per group. Scale bar = 200 μm. Colonic MPO activity (**g**) and spleen weight ((**h**), n = 7–10 spleens per group) were analyzed. n.s. > 0.05, * *p* < 0.05, ** *p* < 0.01, *** *p* < 0.001, **** *p* < 0.0001 as determined by one-way ANOVA (**c**,**e**–**h**). Data represent mean ± SEM.

The intestinal barrier serves as a physical obstacle that prevents the passage of pathogens, toxins, and allergens from the lumen to various tissues, organs, and the blood [22]. This function is crucial in the modulation of the immune system and, consequently, in maintaining overall health [12]. The intricate interplay between structural constituents and molecular interactions within the intestinal mucosa enables it to function dynamically, ensuring the preservation of intestinal integrity and immune homeostasis [12]. As aforementioned, the intestinal microecology was appreciably remodeled in the PS pretreatment groups compared with the non-treated mice (Figure 2c,d), indicating superior efficacy in protecting the biological barrier function. Moreover, it should be noted that *A. muciniphila* became an absolute predominance in the entire microbial community (Figure 2e,f). Intrigued by these results, our attention subsequently shifted towards investigating the impact of PS on the functions of the colonic mucus layer, aiming to enhance the comprehension of its influence on mucosal barrier mechanisms. The results obtained from AB and PAS staining revealed a significant increase in the quantity of goblet cells that produced mucus in the colon following PS treatment when compared with the group that did not receive the pretreatment (Figure 4a). MUC2, the predominant mucin protein responsible for the formation of the mucus layer, underwent extensive glycosylation and was actively secreted by goblet cells [12]. We also extended the detection by immunohistochemistry and found that pretreatment with PS led to a greater area of MUC2^+^ cells compared with the nontreated group (Figure 4b).

The epithelial cells, situated beneath the mucus layer, heavily contribute to the physical integrity of the intestinal barrier [6,7,12]. We next performed Western blotting and immunofluorescence staining and found that the expression of tight junction proteins, such as ZO-1 and occludin, was higher in the PS treatment groups compared with the nontreatment group (Figure 4c,d).

To further understand the protective efficacy of PS, we analyzed the content of colitis-associated inflammatory cytokines and found that pretreatment with PS reduced the levels of pro-inflammatory cytokines, such as IL-1β, TNF-α, and IL-6, while increased anti-inflammatory TGF-β1 cytokine levels (Figure 4e). We also confirmed the differences in immune cells in the intestinal epithelium and lamina propria by immunohistochemistry; although the untreated group exhibited a higher degree of neutrophil infiltration, there was no difference in macrophage infiltration (Figure 4f). Also, compared with the DSS group, PS pretreatment not only resulted in lower colonic IBD-associated myeloperoxidase (MPO) activity [1] but also in less splenomegaly (indication of inflammation severity) (Figure 4g,h). Taken together, these data presented suggest that pretreatment with PS exhibited a greater ability to mitigate damage to the intestinal mucosal and colonic epithelium barrier, as well as reduced intestinal inflammation induced by DSS-colitis, when compared with nontreatment.

### 3.5. The Original Gut Microbiota Mediated the Protective Effect of PS against Colitis Induced by DSS

**Figure 5:**
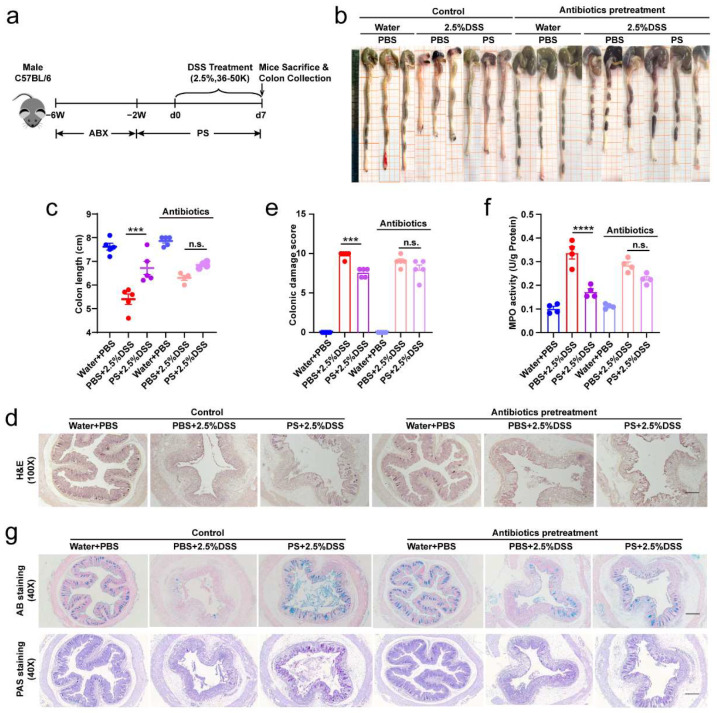
The original gut microbiota mediated the intestinal mucosal barrier enhancement of PS to protect against DSS-induced colitis. (**a**) Schematic showing depletion of the original intestinal flora in DSS-induced colitis mice using broad-spectrum antibiotics (ABX) for 4 weeks. n = 7–9 mice per group. (**b**) Representative colons from each group described in (**a**) and statistical analysis for colon length. (**c**) Representative photomicrographs (100×) of colon tissues staining by H&E ((**d**), scale bar = 200 μm), and quantitative analyses for colonic damage scores (**e**). (**f**) Activity of colonic MPO after ABX depletion of mice described in (**a**). (**g**) Representative photomicrographs (40×) of colon tissue staining by AB and PAS. Scale bar = 500 μm. n.s. > 0.05, *** *p* < 0.001, **** *p* < 0.0001 as determined by one-way ANOVA (**c**,**e**,**f**). Data represent mean ± SEM.

To investigate the effect of intestinal microbiota on colon damage and to determine whether the effect of PS treatment is dependent on the original intestinal microbiota, we used broad-spectrum antibiotics (ABX) to deplete the original gut microbiota for 4 weeks, followed by an additional 2 weeks of pretreatment with oral PS treatment prior to the induction of damage using DSS (Figure 5a). We found that the change in colon length, colonic histological damage, MPO activity, and the number of mucus-producing goblet cells was in accordance with the previous finding above in mice without ABX treatment. Even so, the administration of PS following the elimination of intestinal microbiota using ABX led to a reduced effectiveness of PS in protecting against DSS-colitis, in comparison with the ABX-treated group that did not receive PS supplementation (Figure 5b–g). These findings indicated that the presence of the original intestinal microbiota partially contributed to the protective effect of PS.

### 3.6. PS Protective Effect against DSS-Induced Colitis Involved Inflammation and Immune Function-Associated Signaling Pathways

**Figure 6:**
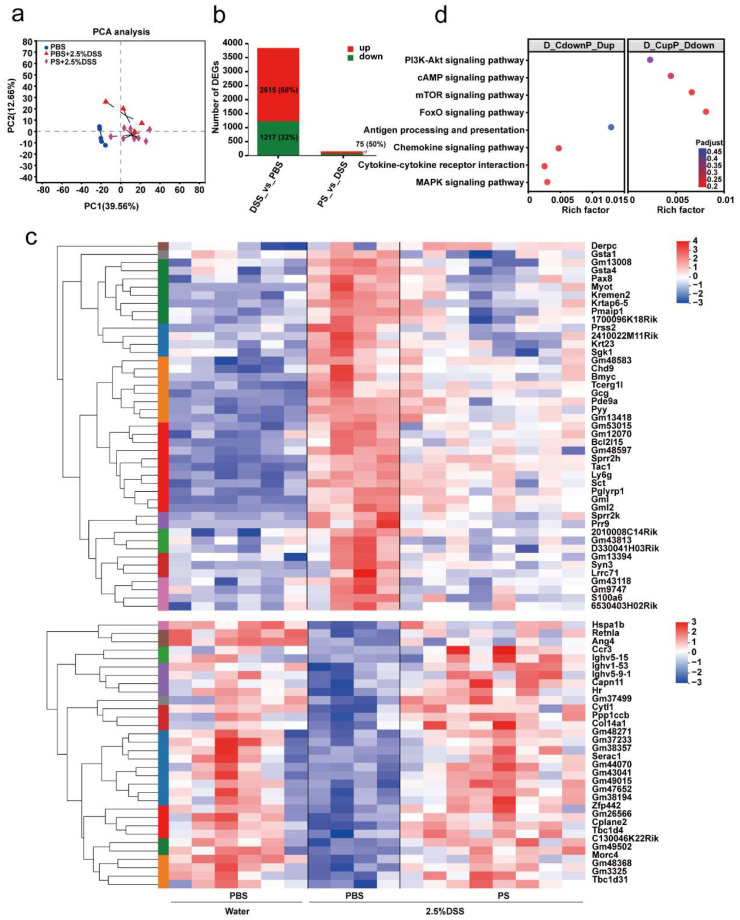
PS protective effect against DSS-induced colitis involves inflammation and immune function-associated signaling pathways. (**a**) Score scatter plot from PCA model of non-ABX-treated colons of mice described in Figure 5a. n = 4–8 mice per group. The black dashed lines represented the lines between samples within the same group (with 95% confidence intervals). (**b**) Quantitative statistics of the number of differential expression genes between any two groups (|log2fold change|≥ 1 & adjust *p* < 0.05). (**c**) Hierarchical clustering heatmap of differential expression genes in each group described in (**a**). (**d**) Bubble plot of selected KEGG enrichment analysis for differential expression genes described in (**c**).

To clarify the fate of colon tissue involved in PS-induced intestinal mucosal barrier protection, we conducted RNA sequencing (RNA-seq) of colon tissue samples dissected from non-ABX-treated groups described in Figure 5a. From the PCA score chart, we found the genes were significantly different across three groups (Figure 6a). Further, we analyzed the significantly differential genes (DEGs) between selected two groups based on the quantification of gene expression (|log2fold change| ≥ 1 & adjusted *p*-value < 0.05, Figure 6b): DSS-induced group versus uninduced group, 3832 DEGs (2615 were upregulated, while 1217 were downregulated); PS pretreatment group versus DSS-induced group, 150 DEGs (75 were upregulated, while 75 were downregulated).

Next, we sought to pinpoint the DEGs that were upregulated in the DSS-induced group compared with the uninduced group but that were downregulated simultaneously in the PS pretreatment group compared with the DSS-induced group; a total of 45 genes were detected (Figure 6c, upper panel); on the contrary, 32 genes were clarified (Figure 6c, lower panel). Subsequently, these DEGs were respectively subjected to KEGG and GO pathway enrichment analysis. A bubble plot showed the signaling pathways and signaling molecule and interaction associated with inflammation and the immune system, including FoxO, mTOR, cAMP, PI3K-Akt, MAPK signaling pathway, antigen processing and presentation, chemokine signaling pathway, and cytokine–cytokine receptor interaction (Figure 6d). Intriguingly, these pathways, signaling molecules, and interaction concentratedly mapped to four genes: *Sgk1*, *gcg*, *ccr3*, and *Hspa1b*, which suggested high multifunctionality. These data suggested that inflammation and immune function-associated signaling pathways were involved in the protective effect of PS against DSS-induced colitis.

## 4. Discussion

In this study, we determined the protective effect of natural bioactive polysaccharides on UC in mice and described that gut microbiota was the crucial event in mediating the prebiotic-like function. Compared with the untreated group, supplementation of the polysaccharides from *N. commune* exhibited a superior capacity to improve intestinal mucosal barrier function, including improving intestinal dysregulation, alleviating intestinal inflammation and reducing inflammatory infiltration, and repairing intestinal epithelial tight junction integrity. We then conducted a cocktail of antibiotics to validate the mediation of gut microbiota. Furthermore, we found that inflammation and immune function-associated signaling pathways were involved in the protection.

UC used to be more common in Western high-income countries, but the epidemiological paradigm has changed over the past two decades, with rates stabilizing in Western countries and increasing substantially in emerging economies [1,2,3]. The pathogenesis of UC is multifaceted and still not entirely understood [1]; but, it has been recognized that UC is an intestinal barrier disease [1] characterized by inflammation confined to the mucosa [1,2,3] and initially caused by either an epithelial cell or structural intestinal epithelial dysfunction [1,6]. As a physical, chemical, immune, and biological barrier, the intestinal barrier prevents pathogens, toxins, and allergens from spreading to other tissues, organs, and blood [22], while allowing nutrients to be absorbed and immune responses to be triggered and limiting the spread of potentially harmful microorganisms and antigens [12]. Few luminal antigens enter the lamina propria when there is a functional intestinal barrier, which is made up of epithelial cells and a mucus layer on top. The immune cells in the lamina propria are prevented from mounting a pro-inflammatory immunological response by the current tolerance mechanisms [1]. These tolerance mechanisms, however, breakdown as the barrier breach widens and more luminal antigens pass through it, leading to the stimulation of local immune cells, the production of chemokines, and the subsequent infiltration of immune cells that worsen the inflammatory process [1,14]. The intestinal barrier assumes a crucial role in the modulation of the immune system and, hence, in health [12,14]. Intestinal barrier dysfunctions have been linked to a diverse array of diseases. The fundamental treatment goal ought to be the preservation of barrier function [1,12], which designates a new therapeutic target [12].

The pathogenesis of UC is complicated; however, there is a close relationship between it and intestinal microbiota [5,32]. Studies have shown that the disruption of resident microbiota may be the major factor in inducing IBD [5]. Probiotics can effectively induce and maintain remission in UC patients, indicating that optimizing intestinal microbiota is the key to treat UC [5,33]. *A. muciniphila*, populating the mucus layer of the gastrointestinal tract, maintains intestinal immunity and regulates gut barrier functions [34]. In this study, we found that the relative abundance of *A. muciniphila* exhibited absolute predominance in the colonic microbiome of mice pretreated with PS, acting as the central differential species (Figure 2e,f). It was therefore reasonable to speculate that *A. muciniphila* improved mucus thickness [34] by regulating ZO-1 and occludin expression (Figure 4a,c). Additionally, *A. muciniphila* reinstated the amount of goblet cells [34] and enhanced both the expression and secretion of MUC2 (Figure 4a,b), accompanied by the reduced level of pro-inflammatory cytokine TNF-α (Figure 4e). Our results also showed that *uncultured_bacterium_g__norank_f__Muribaculaceae* was enriched after the treatment with PS (Figure 2e,f), which was identified as one possible target microorganism in the development of colitis [35]. Previous studies reported that some members of *norank_f_Muribaculaceae* could hinder the growth and colonization of pathogenic bacteria in the intestine by occupying ecological space [36]. We also observed a notable increase in *unclassified_g__norank_f__norank_o__Clostridia_UCG-014* in the gut microbiota of PS-treated mice (Figure 2e,f). *Clostridia_UCG-014* has commonly been reported as a pro-inflammatory bacterium, but it has also been reported to reduce in UC [37], indicating that the multiple members of *Clostridia_UCG-014* may play different roles in the gut. The relative abundance of *norank_f_norank_o_Clostridia_UCG-014* has been shown to increase during the first 7 days post-UC and then decrease [38], consistent with our results harvested on the 10th day post-DSS induction.

One of the major functions of the gut microbiota is the digestion of dietary components and the production of active metabolites to directly affect human physiology including gut barrier function [15,16,17]. Here, we also examined the changes by metabolomics; the KEGG pathway enrichment analysis showed the differential metabolites (PCs and TXB 2) mapped to arachidonic acid (AA) metabolism (Figure 3b,c). AA and its metabolites have attracted a lot of attention in cardiovascular and cancer biology [39], particularly concerning inflammatory processes and disease (Figure 3c). PC, a typical eukaryotic membrane phospholipid, is present in only about 15% of all bacterial species [40]. However, relatively little has been reported about the relation of PC in colitis and gut barrier function. The most intriguing aspect of PC formation in bacteria lies in its critical involvement in symbiotic and pathogenic microbe–host interactions [40]. In some organisms, the absence of PC also interferes with motility, bacterial growth, and stress response [40], indicating that PC plays an important role in infection and inflammation (Figure 3d). Spearman correlation analysis in Figure 3e showed that *uncultured_bacterium_g__norank_f__Muribaculaceae* exhibited a significantly negative correlation with the level of all PCs (*R* < −0.54, *p* < 0.01). We can speculate that *uncultured_bacterium_g__norank_f__Muribaculaceae* could inhibit the growth and colonization of PC-producing bacteria and then occupy ecological space. TXB 2 is the stable metabolite of TXA 2, which possesses pro-inflammatory action in AA metabolism [41]. The generation of TXA 2 can be estimated by the measurement of TXB 2. Accordingly, we observed a significant reduction in TXB 2 level after PS intervention obtained from the colonic content of mice (Figure 3e) and was significantly and negatively correlated with *unclassified_g__norank_f__norank_o__Clostridia_UCG-014* (Figure 3e).

It should be recognized that currently available treatments for UC, including aminosalicylic acid, corticosteroids, immunomodulators, monoclonal antibodies, and even surgeries, often have side effects, drug resistance, and poor clinical efficacy [18,19]. Natural bioactive polysaccharides have attracted increasing attention for their exceptional advantages of safety, accessibility, and good biocompatibility [19,21]. It is well established that most of them can be metabolized by the gut microbiota and can exert profound protection against oxidative stress, toxins, inflammatory response, and tumors [22]. Although the protection of natural polysaccharides against colitis has been recently extensively studied, the role of gut microbiota and gut microbiota-derived metabolite remains incompletely understood and confirmed. The polysaccharides from *N. commune* have some favorable characteristics, including being identified as a heteropolysaccharide with an ultra-high molecular weight of 210 kDa, as well as accounting for up to 60% of the dry weight of *N. commune* [24], strongly implying fairly compelling bioactivity. In this study, we conducted a cocktail of antibiotics to deplete intestinal microbiota from mice for 4 consecutive weeks using a combined low dose of antibiotics for maintaining continuously, thus demonstrating intestinal microbiota-mediated protective effects of polysaccharides from *N. commune* on colitis (Figure 5).

The damage to the structure and function of the intestinal epithelial cell barrier can disrupt the balance in the mucosal immune system and trigger an inflammatory response, exacerbating the damage to the intestinal epithelial barrier [6,22]. Cytokines are crucial targets for inflammation management and play a key role in T cell differentiation and regulation [22]. TNF-α is a potent pro-inflammatory mediator implicated in intestinal barrier defects [12], which frequently collaborates with IL-1β and IL-6 to enhance the inflammatory response by promoting the recruitment and activation of other inflammatory elements, resulting in increased production and release of inflammatory mediators [22] (Figure 4e). The gut is the primary metabolic site for polysaccharides, while the ultimate fate of it regulated by polysaccharides is always fascinating. Transcriptomics analysis showed that differential genes of colon tissue from mice in each group enriched in inflammation and immune system-associated signaling pathway and signaling molecule and interaction, including FoxO, mTOR, cAMP, PI3K-Akt, MAPK signaling pathway, antigen processing and presentation, chemokine signaling pathway, and cytokine–cytokine receptor interaction (Figure 6d). The development of IBD is attributed to polygenic and multifactorial pathology via activation of certain signaling pathways [4]. In IBD pathophysiology, aberrant signaling pathways cause deregulation of the inflammatory response. The IBD-related signaling pathways mainly include NF-κB, MAPK, JAK/STAT, and PI3K/TLR4 signaling pathways [4]. Of these, the PI3K pathway is a cell-surface-receptor-controlled signal transduction pathway that is involved in the regulation of important leukocyte processes, such as activation, proliferation, and recruitment [4]. PI3K can regulate the anti-apoptotic signals and pro-inflammatory signals triggered by LPS and TNF-α [4]. Disruptions of signaling pathways act on the intestinal barrier, resulting in the unrestricted release of pro-inflammatory cytokines [4]. Therefore, we are prone to speculate that polysaccharides of *N. commune* could inhibit the expression of pro-inflammatory cytokines and protect the damaged intestinal barrier by regulating inflammation and immune function-associated signaling pathways (Figure 6d).

In summary, our work integrated multi-omics profiling to reveal that the polysaccharides from *N. commune* predominantly enhanced the intestinal microbiota-mediated intestinal mucosal barrier to confer protection against UC in mice and exhibited dramatical prebiotic-like functions, providing an alternative or complementary treatment for IBD, especially UC.

## Data Availability

Data will be made available on request.

## Supplementary Materials

The following supporting information can be downloaded at: https://www.mdpi.com/article/10.3390/nu15133054/s1, Figure S1: Relative abundance of gut bacterial phylum in each group as described in Figure 2a. Figure S2: The Venn diagram of termed metabolic sets (upper) and the number of differential metabolites between each PS treatment group and DSS-induced group (lower).
