# Supplementary material for: Potent Intestinal Mucosal Barrier Enhancement of Nostoc commune Vaucher Polysaccharide Supplementation Ameliorates Acute Ulcerative Colitis in Mice Mediated by Gut Microbiota
Source: Nutrients. 2023 Jul 6;15(13):3054. doi: 10.3390/nu15133054 (PMC10346458; doi:10.3390/nu15133054)
Supplement: Supplementary file 1 [file nutrients-15-03054-s001.zip › nutrients-2474899-supplementary.pdf]

Supplementary Figures

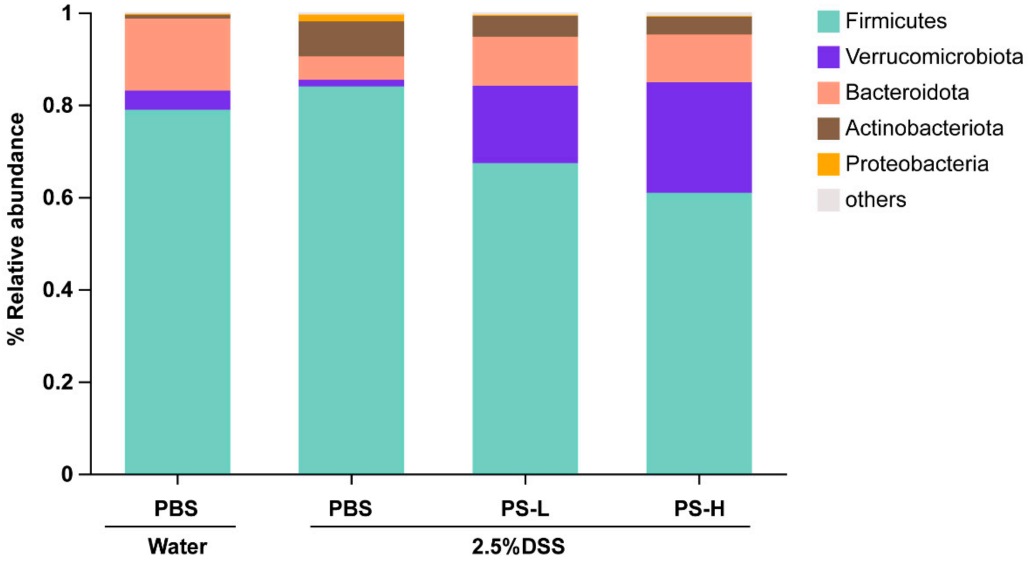

Supplementary Figure S1. Relative abundance of gut bacterial phylum in each group as described in Figure 2a.

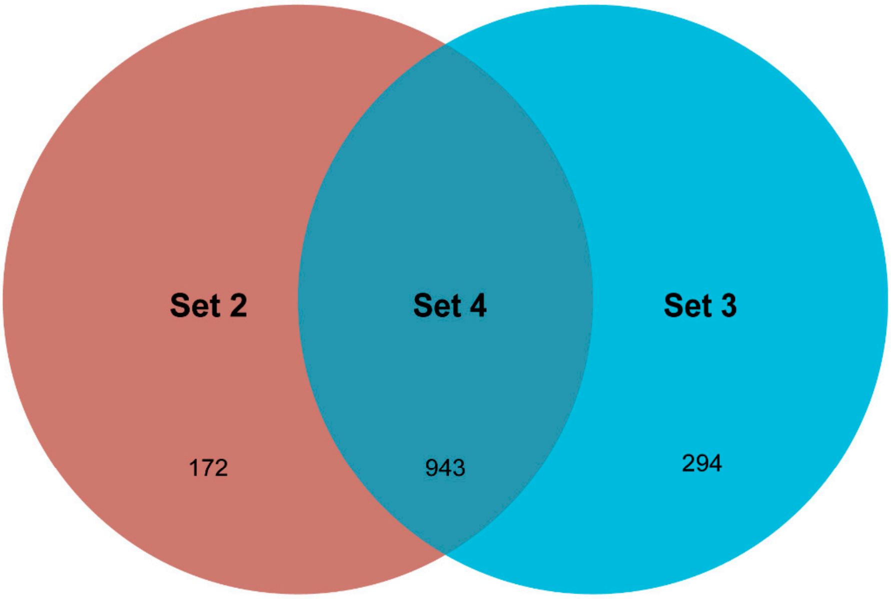

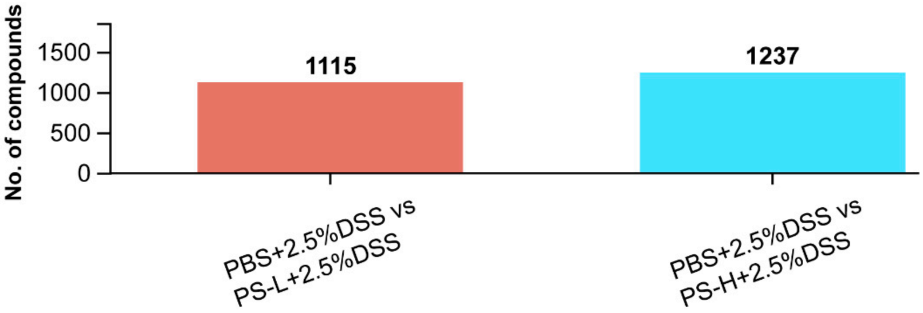

Supplementary Figure S2. The Venn diagram of termed metabolic sets (upper) and the number of differential metabolites between each PS treatment group and DSS-induced group (lower).
